# Distinct mutations emerge in the genome of serotype O foot-and-mouth disease virus during persistence in cattle

**DOI:** 10.1128/jvi.01422-24

**Published:** 2025-02-07

**Authors:** Benedikt Litz, Leonie F. Forth, Florian Pfaff, Martin Beer, Michael Eschbaumer

**Affiliations:** 1 Institute of Diagnostic Virology, Friedrich-Loeffler-Institut 39023 , Greifswald-Insel Riems, Germany; Emory University School of Medicine, Atlanta, Georgia, USA

**Keywords:** FMDV, persistence, carrier, sequencing, mutation, SNP

## Abstract

Like other RNA viruses, foot-and-mouth disease virus (FMDV) has a high mutation rate. After the acute phase of infection, about half of infected cattle develop a persistent FMDV infection that can last for weeks or months. During this persistent phase, the virus continues to replicate, resulting in the emergence of genomic heterogeneity. We have documented the pattern of mutations in the persistent phase by obtaining consensus-level sequences directly from oropharyngeal fluid (OPF) without prior virus isolation in culture. OPF samples were repeatedly collected from 22 experimentally infected cattle, 20 of which were virus positive in the OPF on day 21 after infection or later. We observed that during the persistent phase, the amount of non-synonymous mutations causing an amino acid change increased over time. Two amino acid changes that showed a striking increase during the persistent phase, VP3 A75T and VP2 Y79H, were present neither in the inoculum nor during the acute phase. Another amino acid change in VP3, R56C, which was previously implicated in FMDV pathogenicity, was already present in the inoculum and dominated toward the end of the trial in most samples. Several other amino acid changes occurred, particularly on the surface of VP2 around residue VP2 79. By functional analysis, we show that the persistent isolates evolve distinctly compared with cell culture adaptation but do not show signs of antigenic escape from neutralizing antibodies. In agreement with previous observations, we conclude that these amino acid changes are indeed associated with persistent infection of cattle with FMDV serotype O.

**IMPORTANCE:**

Our research article describes the genetic changes that occur during the acute and persistent foot-and-mouth disease (FMDV) infection. This is of particular interest to understand viral dynamics within an infected population from which new viral strains could emerge. Especially FMDV, with its high antigenic diversity and very limited cross-reactivity between strains and serotypes, has already demonstrated in the past that new variants can quickly emerge and evade vaccine responses. In our study, we have observed that this dynamic evolution continues during the persistent phase. Persistently infected animals, which are clinically indistinguishable from healthy animals, also pose a reservoir for recombination. A better understanding of viral dynamics is essential for improved vaccines to prevent the emergence of antigenic variants.

## INTRODUCTION

RNA viruses have developed a distinct evolutionary strategy by maintaining a high error rate of their RNA-dependent RNA polymerase. The naturally occurring virus population is therefore, in some cases, composed of a viral swarm or quasispecies, which supports rapid adaptation to changing environments (1). For foot-and-mouth disease virus (FMDV, species *Aphthovirus vesiculae*), which belongs to the genus *Aphthovirus* in the family *Picornaviridae*, the high mutation rate has resulted in a diverse set of seven serotypes with many subtypes. The mutation rate is estimated to be 7.8 × 10^−4^ per nucleotide and transcription event, which equals approximately one mutation per genome per replication cycle (2). In comparison, severe acute respiratory syndrome coronavirus 2 (SARS-CoV-2) has a mutation rate of 1.3 × 10^−6^ per nucleotide and infection cycle (3). Eradication measures against FMD, a highly infectious transboundary disease of livestock, are impeded by the limited cross-reactivity between newly emerging strains and available vaccines, due to the high genetic and antigenic diversity of FMDV (4).

The FMDV genome codes for one large open reading frame (ORF), which is translated into a single polyprotein. This polyprotein contains the precursor of four structural proteins (VP1, VP2, VP3, and VP4) and eight non-structural proteins (L^pro^, 2A, 2B, 2C, 3A, 3B^1-3^, 3C, and 3D). The structural proteins form the capsid, with VP1, VP2, and VP3 exposed on the outer surface, while VP4 is located on the inside of the capsid (5). Therefore, the structural proteins on the outside are major targets of neutralizing antibodies, while VP4 is hidden from the humoral response. The capsid surface features five antigenic sites, which influence the serotype and cross-reactivity. The “antigenic site 1” on VP1 has long been considered to be immunodominant and stood in the focus of research, but following vaccination against serotype O, the strongest antibody response was directed against “antigenic site 2” on the surface of VP2 (6). A peculiarity of “site 1,” however, is the G-H loop of VP1 with an RGD-binding motif, which facilitates the binding to integrin receptors as a first step in viral cell entry. Apart from its antigenicity and the receptor-binding domain, the G-H loop of VP1 is motile and its orientation can influence its antigenicity (7).

FMDV causes a highly infectious disease in cloven-hoofed animals characterized by vesicular lesions in the mouth, on the muzzle, and in the interdigital cleft. In about 50% of FMDV-infected cattle, the virus remains in the cattle after the acute phase and persistently infects the epithelia of the nasopharynx. These persistently infected animals are called “virus carriers.” The persistent phase of infection follows the acute phase characterized by clinical signs of FMD and usually resolves after 10 days postinfection (dpi). The animal then enters a transitional stage in which virus is either cleared from the nasopharynx or remains persistent (8). An infection that lasts longer than 28 dpi is conventionally defined as a persistent infection, while the earliest time point for identifying persistently infected cattle is as early as 21 dpi (9). Even though infectious virus can be recovered from persistently infected cattle by sampling oropharyngeal fluid (OPF) with a probang cup, which collects superficial cells and saliva from the pharynx (10), it is still debated whether persistently infected cattle are contagious at all (8, 11).

The preferred sites of persistent infection are the epithelia of the nasopharynx, namely the dorsal nasopharynx (DNP) and the opposing dorsal soft palate (DSP). Here, the follicle-associated epithelia (FAE), which covers the mucosa-associated lymphoid tissue (MALT), are infected (9). The patchy distribution of the MALT in the pharynx results in a very focal occurrence of persistent infection (12). Because this remote location is not easily accessible by standard sampling techniques, use of the probang cup was established early in FMDV research. Using this metal cup, saliva and epithelial cell scrapings are collected from the pharynx, the so-called oropharyngeal fluid (OPF), from which virus can be recovered if the animal is persistently infected (13).

In Theiler’s virus (species *Cardiovirus theileri*), a picornavirus causing infection of the central nervous system in mice, a single amino acid change is responsible for the persistent virus phenotype (14); hence, studies of virus isolates from the persistent phase of FMDV were focused on finding similar mutations. Historically, sequencing was focused on VP1, as performed by Gebauer et al., who claimed that the number of amino acid substitutions fixed in VP1 during the persistent phase was similar to that after several years of acute-phase transmission (15). Other studies found certain amino acid changes to be associated with persistent infection (16, 17), but these were observed to also occur during acute transmission and were not considered unique to persistent replication (18). More recent investigations using whole-genome sequencing focused on the mechanisms of virus evolution during the persistent phase (19, 20). They showed that early during the acute phase, several haplotypes, which were acquired in the infection, can coexist, but over time haplotypes with new mutations become increasingly dominant. During the persistent phase, multiple viral subpopulations within an animal could still be distinguished, reflecting the focal nature of infection.

We sequenced the entire ORF of FMDV from 20 persistently infected animals directly from OPF without prior amplification in cell culture. To our knowledge, this is the largest set of native whole-ORF sequences from experimentally infected carriers collected so far. Samples were collected in the acute, transitional, and persistent phases. This allowed us to document the evolution of FMDV and its adaptation to the host.

## RESULTS

### Animal trials

In the vaccination study, animals vaccinated with the experimental vaccine were not protected from challenge infection and developed clinical signs with vesicular lesions similar to those observed in non-vaccinated animals. The commercially vaccinated cattle did not develop clinical signs. However, the efficacy of the vaccines and the outcome of the challenge infection are outside of the scope of this paper. An overview of clinical data collected in the vaccination study is shown in Fig. S1. In the infection study, all animals developed clinical signs consistent with FMDV.

### Variation in the inoculum

For the vaccination trial, the plaque-purified clone of FMDV O/FRA/1/2001 was passaged once in a heifer. Lesion material from the tongue was collected and deep sequenced to examine the consensus sequence and minor variants. The consensus sequence remained unchanged in comparison with the plaque-purified clone (GenBank accession OV121130); however, 50 minor variants were detected with a frequency between 0.5% and 22% (Table S1). Of the 50 minor variants, 33 were in the coding region, of which 25 were non-synonymous variants. Of the 33 variants in the ORF, 25 variants were in the P1 region, with three variants in VP4, six variants in VP2, 10 variants in VP3, and six variants in VP1. In some cases, two variants were found at the same position (e.g., positions 2352 and 2354 corresponding to residue 134 of VP2, positions 2772 and 2773 corresponding to residue 56 of VP3; Table S1). Neighboring variants were not present on the same read and are therefore likely to represent independent genomic variants. From the non-synonymous variants, seven variants rose to consensus level at a later time point of the trial. Majority of these are located in the capsid-coding region. The minor variant with the highest frequency in the inoculum, VP3 R56C (22.1%), later became dominant in the vast majority of consensus sequences recovered throughout the whole study, from the acute phase as well as from the persistent phase of infection. Not all variants were detected throughout the study. Missense variants like VP3 R56L, VP1 H195L, and VP1 H195Q were detected at the consensus level in samples from the acute phase but were absent in OPF samples from 10 dpi onward. VP3 G60D was present in the inoculum at 2% and detectable at the consensus level in samples from the acute phase. It then disappeared from the consensus sequences, only to reappear at 35 dpi in animal 509. Another missense variant was absent in the acute phase but emerged during persistent infection: 3A S140F, which was found in the consensus sequences of two animals (ID 943 and 946) from 14 and 17 dpi, respectively, onward until the end of the study as well as a polymorphic variant in animal 506 at 28 dpi. VP1 A199D was also present in the inoculum and re-appeared in animal 649 during persistence at 28 and 35 dpi. The variants VP3 56, VP1 195, and 199 are known to be associated with heparan sulfate (HS)-binding sites (21, 22).

In a comparison with field isolates from a large outbreak caused by a closely related virus in the United Kingdom in 2001 (23), six amino acids were different in our inoculum: L^pro^ I104V, VP3 H56R, VP1 E198G, 3A I3S, 3D A94T, and 3D F294I.

### Variation in vesicular lesions from the acute phase

Deep sequencing of vesicular material from six animals collected during the acute phase revealed changes in the consensus sequence in each sample, including one to three variants fixed in the consensus sequence and four to nine minor variants in the coding region compared with the inoculum. In animal 191, variants at nucleotide positions 2772 and 2773 were present at frequencies of 50.2% and 49.3%, respectively, encoding VP3 R56C and R56H, with only the first represented in the consensus sequence. These two variants were not found on the same reads, likely representing uncorrelated mutations.

Four minor variants from the inoculum were also found in these samples: VP3 R56C, VP3 R56H, VP3 G60D, and VP1 H195Q. Of these, VP3 R56C was dominant in three animals and was present as a minor variant in two others but was not detectable in the sample from one animal (ID 509). Additionally, three minor variants from the vesicular material were detected at the consensus level in other samples from the acute phase of the same animal but not during the persistent phase (2A F4S, 2C P220L, 3D H378T). Interestingly, 2A F4S and 3D H378T were detected in samples from the acute phase of one other animal.

### Incidence of persistent infection and probang samples

Persistently infected animals were defined by positive virus isolation from the OPF collected by probang cup after 28 dpi (24). In the vaccination trial, virus was isolated from 17 of 20 infected animals after 28 dpi, and in one additional animal (ID 940), virus was recovered from the OPF at 24 dpi but not at 28 or 35 dpi. In vaccinated animals, the carrier state can be reliably determined as early as 10 dpi (8); therefore, we included animal 940 in the group of persistently infected animals in further studies. With this animal included, the incidence of persistent infection is 90% (with a confidence interval of 70%–97%). From the two animals, 426 and 662, no virus was recovered from the OPF at any time. Animal 426 had received the commercial vaccine and was clinically protected, but animal 662 had received the experimental vaccine intranasally and developed clinical FMD.

**Fig 1:**
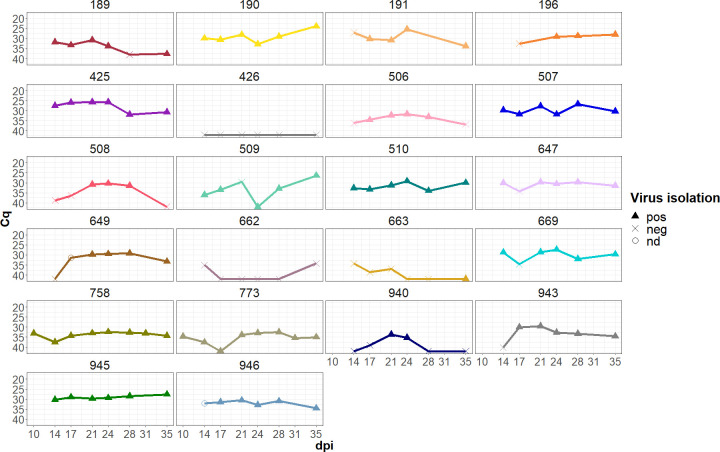
FMDV RT-qPCR results for OPF collected by probang cup starting at 10 or 14 dpi. The results of the virus isolation from the same sample are indicated by the shape of each data point (pos, positive virus isolation/CPE; neg, negative virus isolation/no CPE; nd, not done).

The virus isolation results are consistent with the FMDV RT-qPCR data for the OPF as shown in Fig. 1. There is no definite Cq (quantification cycle) threshold above which virus isolation from the OPF is not possible, but above a Cq value of 35, the rate of positive virus isolations decreases and few positive results are obtained for such samples. Overall, the amount of detectable FMDV RNA often fluctuates between sample days. The overall mean Cq value was 31.8. This is significantly lower than the amount of FMDV RNA detected in nasal fluid during the acute phase (data not shown). The highest FMDV RNA content in the OPF, corresponding to a Cq of 24, was detected in a sample from animal 190 collected at 35 dpi.

### Nucleotide sequence data from the acute and persistent phases

For this study, we analyzed 130 consensus-level sequences spanning the FMDV ORF from 21 animals. A complete sequence of the ORF was obtained for 97 persistent-phase samples from 18 animals. From the acute phase of infection, one serum and one nasal fluid sample collected between 3 and 6 dpi from 16 animals were sequenced. From animals 758 and 773, an additional saliva sample was sequenced. In the clinically protected animals, low levels of viral RNA were detectable in some acute-phase samples by RT-qPCR, but it was too low to be sequenced. For probang samples, the threshold for recovering a full-length ORF sequence was estimated to be around a Cq of 35, which is similar to the viral load necessary for successful virus isolation mentioned above.

### Virus phylogeny

**Fig 2:**
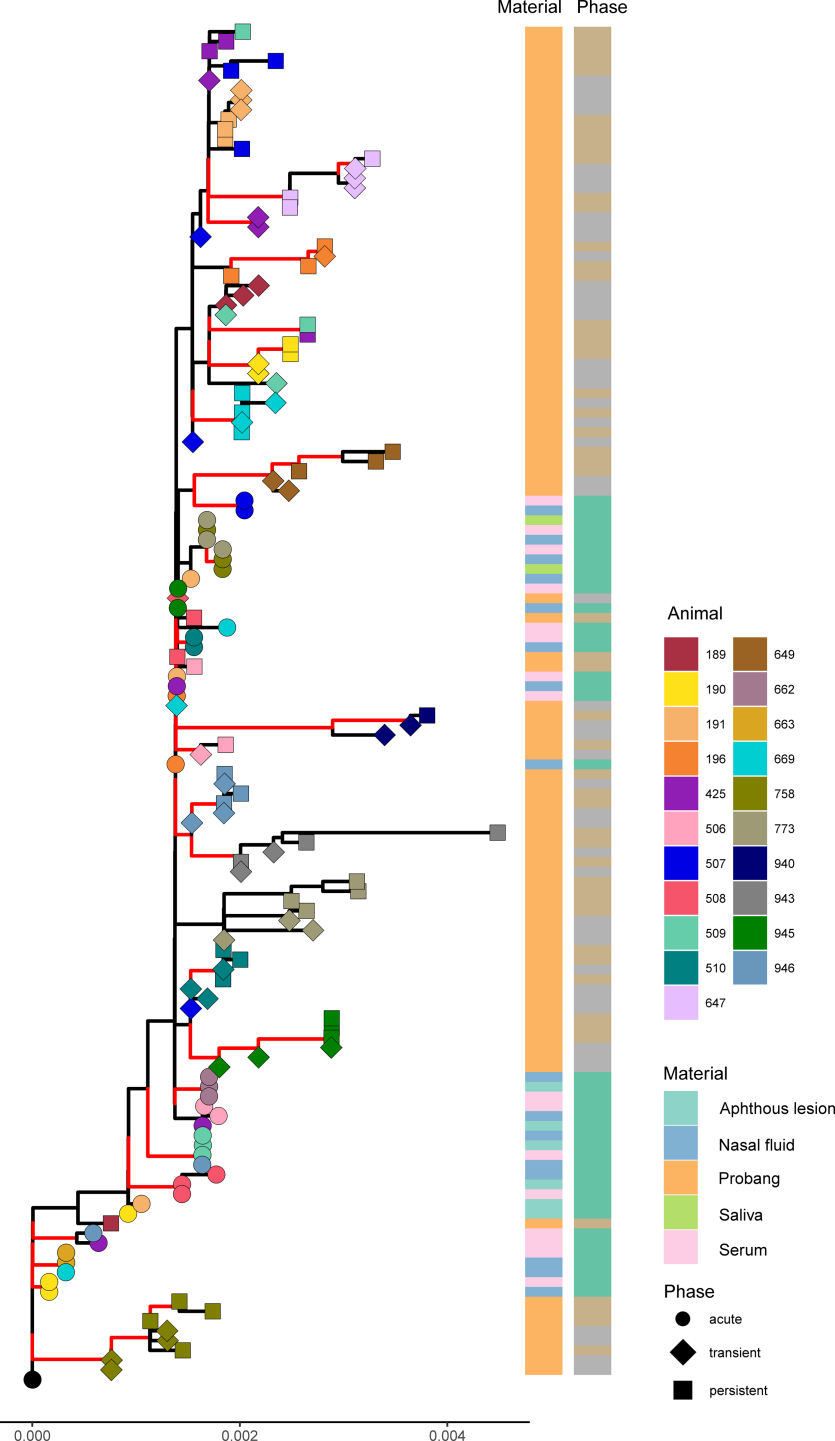
Inoculum-rooted phylogenetic tree of the sequences obtained from cattle persistently infected with FMDV, categorized by animal, sampling material, and phase (acute-phase samples were collected from 3 to 6 dpi, transient-phase samples were collected from 10 to 21 dpi, and persistent-phase samples were collected after 21 dpi). All samples from the same animal are displayed in the same color in the tree. Branches are color coded according to their bootstrap support: ≥95% red; <95% black.

For a phylogenetic analysis to assess the evolution of FMDV over the course of the infection, the sequences were categorized into three phases: the acute phase for samples collected between 3 to 6 dpi, the transitional phase for OPF collected between 10 and 21 dpi, and the persistent phase for OPF collected later than 21 dpi. In the phylogenetic tree, samples appeared to cluster based on the phase of infection. Samples from the acute phase formed two separate clusters. Also, sequences from the transient and persistent phases clustered closely together (Fig. 2). Transient- and persistent-phase samples within the same animal tended to group together. In terms of infection progression, we did not observe a clear phylogenetic trend for OPF samples from later phases of infection to segregate from samples from earlier phases. In some animals, such as ear tags 758 and 773, this seemed to be the case and suggests an accumulation of mutations during infection, but in others (e.g., ear tags 196 and 669), no definite clusters differentiating OPF sequences from the transient and persistent phase can be distinguished.

**Fig 3:**
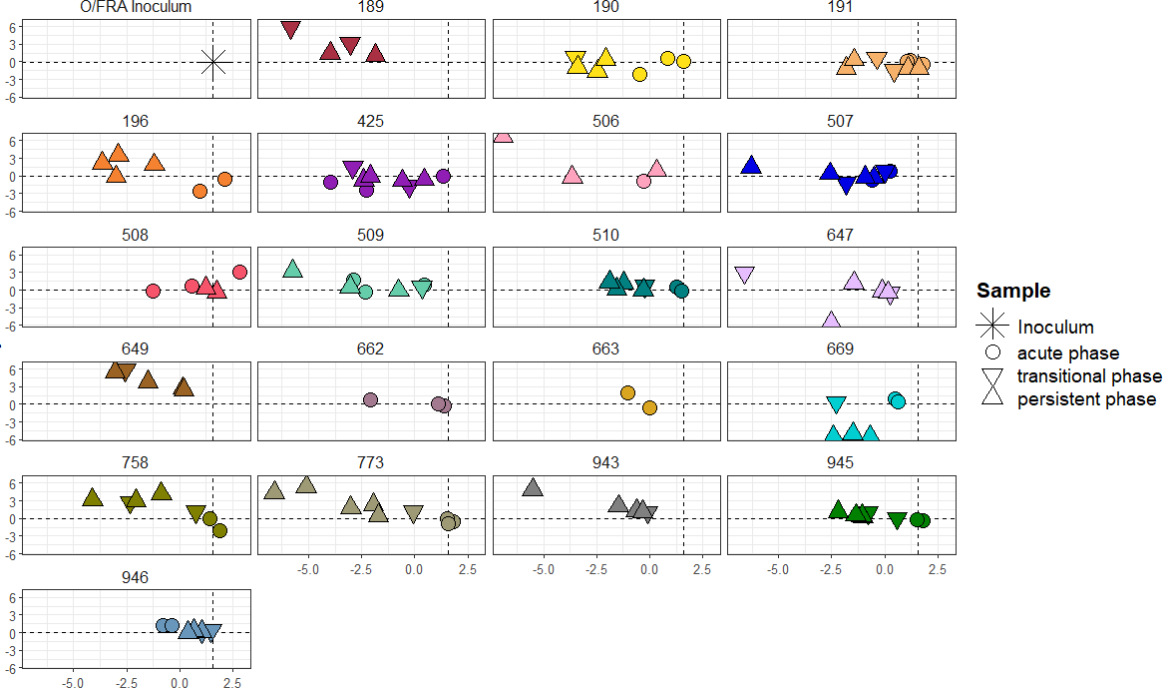
Scaled interpretation of a distance matrix based on the sequences obtained from cattle persistently infected with FMDV (acute-phase samples were collected from 3 to 6 dpi, transient-phase samples were collected from 10 to 21 dpi, and persistent-phase samples were collected after 21 dpi). The inoculum as the origin is indicated in each panel by the intersection of a vertical and a horizontal dashed line. Thirteen samples were excluded from the detailed view.

However, in terms of infection progression, they are based on only a few or even single mutations in the sequences, and the construction of a phylogenetic tree may be compromised by this. Therefore, a classical multidimensional scaling was performed based on the sequence alignment. Distances between each sample were calculated based on the number of different nucleotides between each sequence, and the distances were calculated between them in two dimensions by multidimensional scaling. The distances between the samples were illustrated in a scaled interpretation of this distance matrix (Fig. 3). The findings therein support the results from the phylogenetic tree, which show a close relationship of acute-phase samples to the inoculum and clustered probang samples from the persistent phase of each animal, but still there are some outliers. We did not observe a distinct cluster of samples from the late phase of persistent infection separated from earlier samples from the transitional phase.

### Cell culture passage influences FMDV sequences

**Fig 4:**
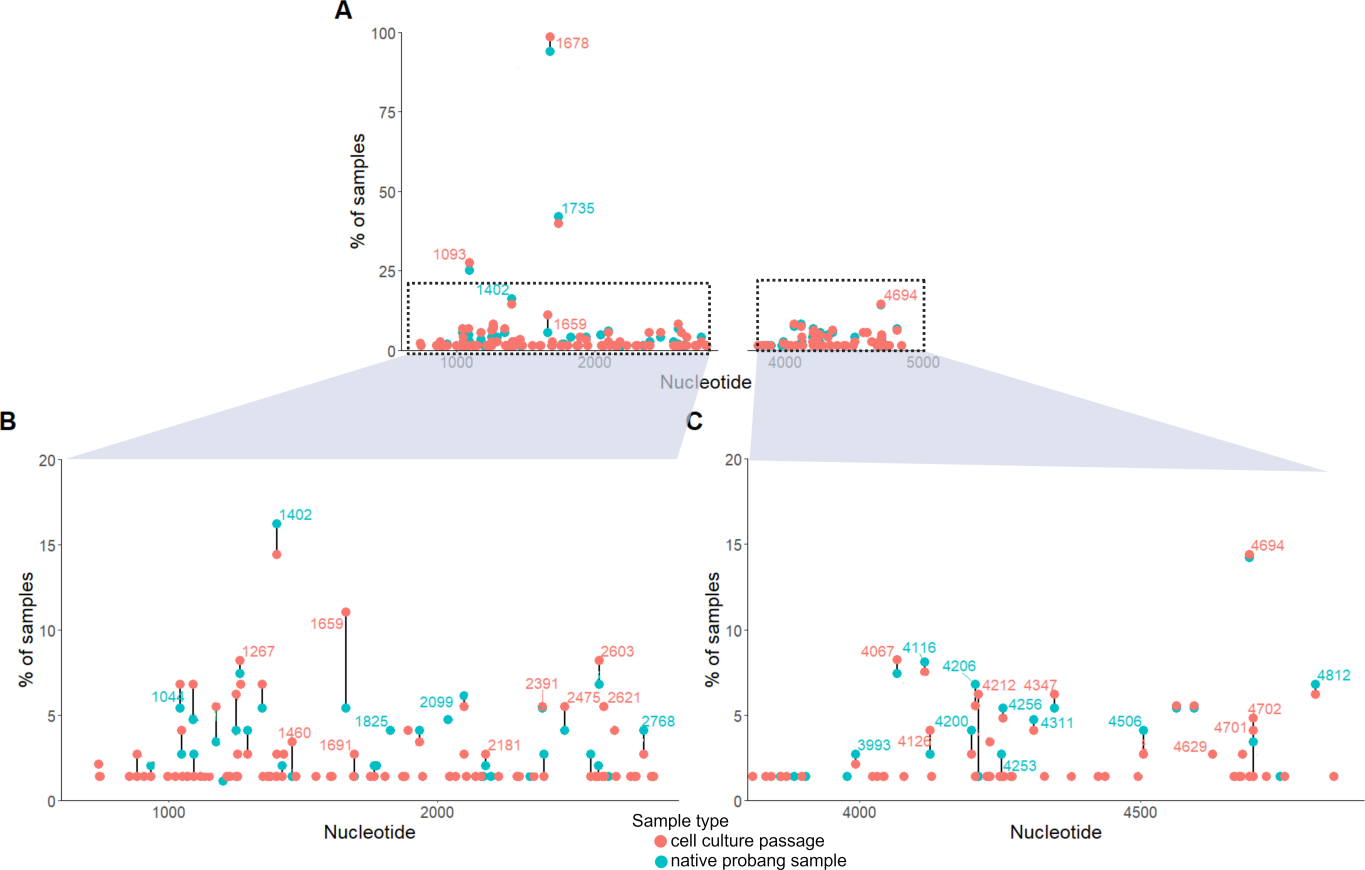
Comparison of mutation frequencies in sequences obtained directly from native OPF collected by probang (blue) and after one cell culture passage (red). Two parts of the genome were included in the comparison, from Lpro to VP3 (nucleotides 600–2900, left) and from 2C to 3B3 (3800–4900, right). (**A**) Overview of all mutations in the compared regions; the gap on the *x*-axis indicates where no sequencing was performed. (B and C) Detailed view of the data from panel **A**. (**B**) Only shows mutations between nucleotides 600 and 2900 and with a frequency of less than 20%. (**C**) Shows only mutations between nucleotides 3800 and 4900 and with a frequency of less than 20%. The comparison only includes samples for which a pair of corresponding sequences (cell culture and native probang) was available.

We compared persistent-phase sequences that were obtained directly from the *ex vivo* OPF sample (probang) with sequences obtained from the same OPF samples after one passage in LFBK-αVβ6 cells, as sequencing of persistent FMDV is usually performed after a cell culture passage because of low viral loads in the OPF (15, 19). The sequences obtained from the virus isolates were in two parts and did not cover the complete ORF; therefore, the comparison only includes Lpro to VP3 and the 3′ half of 2C, 3A, and 3B1-3. The phylogenetic tree for the culture-passaged samples showed a higher degree of sample diversification (data not shown). Some samples from the same animal still clustered together, but more samples from the same individual were scattered across the tree, clustering with sequences from other animals. A side-by-side comparison of the sequences from the culture-passaged samples with directly sequenced samples shows that the mutations found in them were different (see Fig. 4). Generally, more mutations in comparison with the inoculum were present in sequences from the culture-passaged samples (in this set: 76 variants in sequences from the native OPF and 151 variants in the sequences after cell culture passage). New mutations appeared after the passage, and in a few samples, mutations that were present in directly sequenced samples could no longer be detected after passaging. This included the variation at nucleotide position 2040 (amino acid VP2 31), which was present in seven sequences from the native OPF as heterozygotes (ambiguous base call in the Sanger sequencing; counted as 0.5 for the *y*-axis of Fig. 4) but not detectable in the corresponding cell culture samples. Most of the mutations that were introduced by a single cell culture passage were unique and only detected in one sequence. In general, most mutations found in sequences from the native OPF samples were preserved in the corresponding culture sequences but to a varying degree. The highest difference between mutations observed in the native and cell-culture-passaged virus was found at nucleotide position 1659 (amino acid L^pro^ 190). Only in half of the samples that contained a variation at this site in the native OPF was the variation preserved in the cell culture passage.

### Mutations during the acute phase

The consensus sequences of the six vesicular samples and 33 Sanger-sequenced samples from the acute phase (serum, nasal fluid, and saliva) were mapped against the consensus sequence of the inoculum. Herein, we observed 27 mutations, of which 10 were missense mutations. In more than half of the samples (59%) from 11 animals, VP3 R56C was dominant in the consensus sequence. Only three of the acute-phase mutations were later seen in samples from the persistent phase: VP3 R56C, VP3 G60D, and a synonymous mutation at nucleotide 3971 corresponding to amino acid residue 7 of 2B. A detailed breakdown of amino acid changes by animal and sample is given in Table S2.

### Mutations during the persistent phase

**Fig 5:**
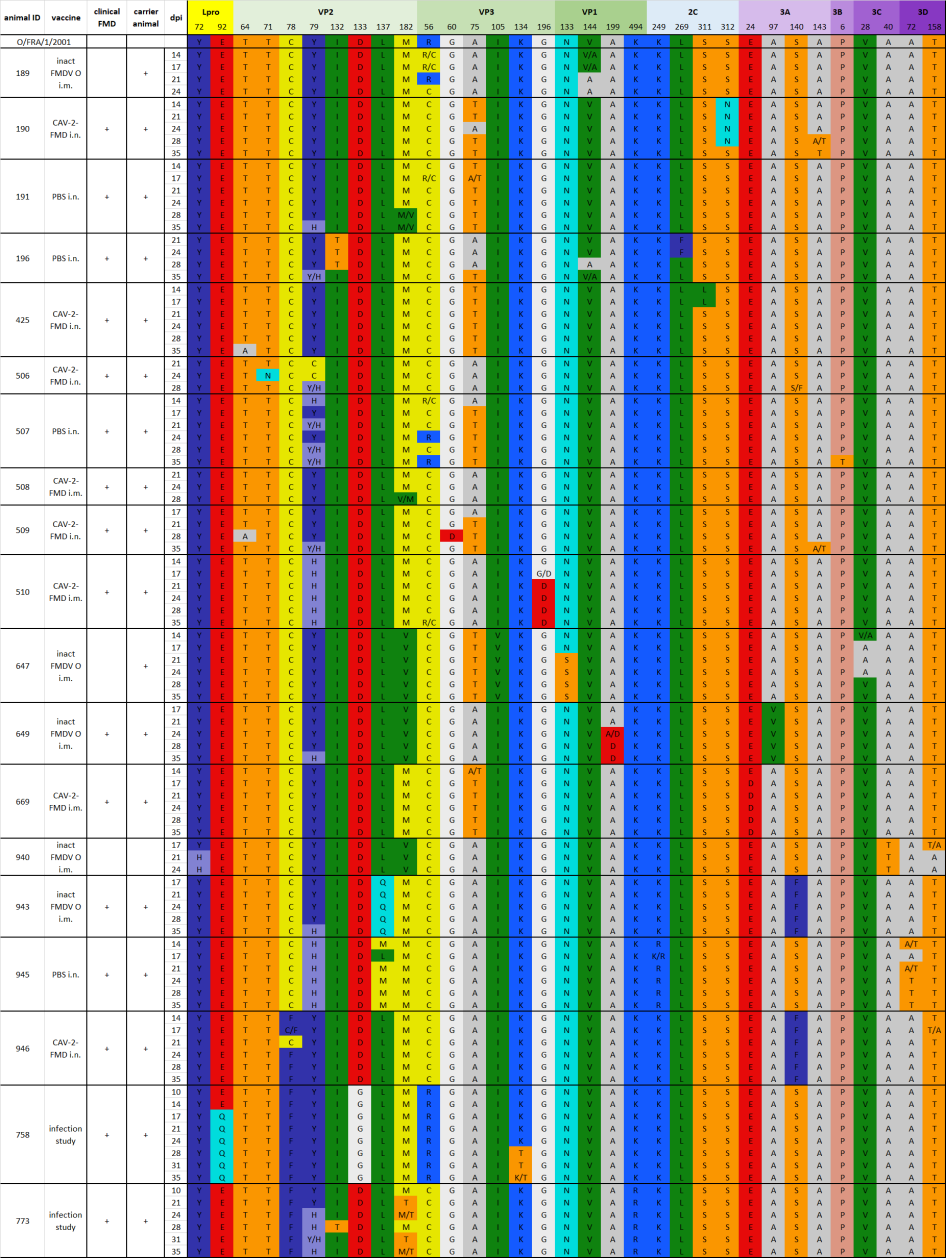
Table of all amino acid changes in sequences recovered from OPF of persistently infected animals starting at day 10 or 14 postinfection. For reference, the consensus sequence of the inoculum is shown at the top. Only sequences with good coverage are listed. The amino acid residues are colored using the RasMol color scheme. The genome position is shown with respect to the reference sequence of the inoculum O/FRA/1/2001 (OV121130).

**Fig 6:**
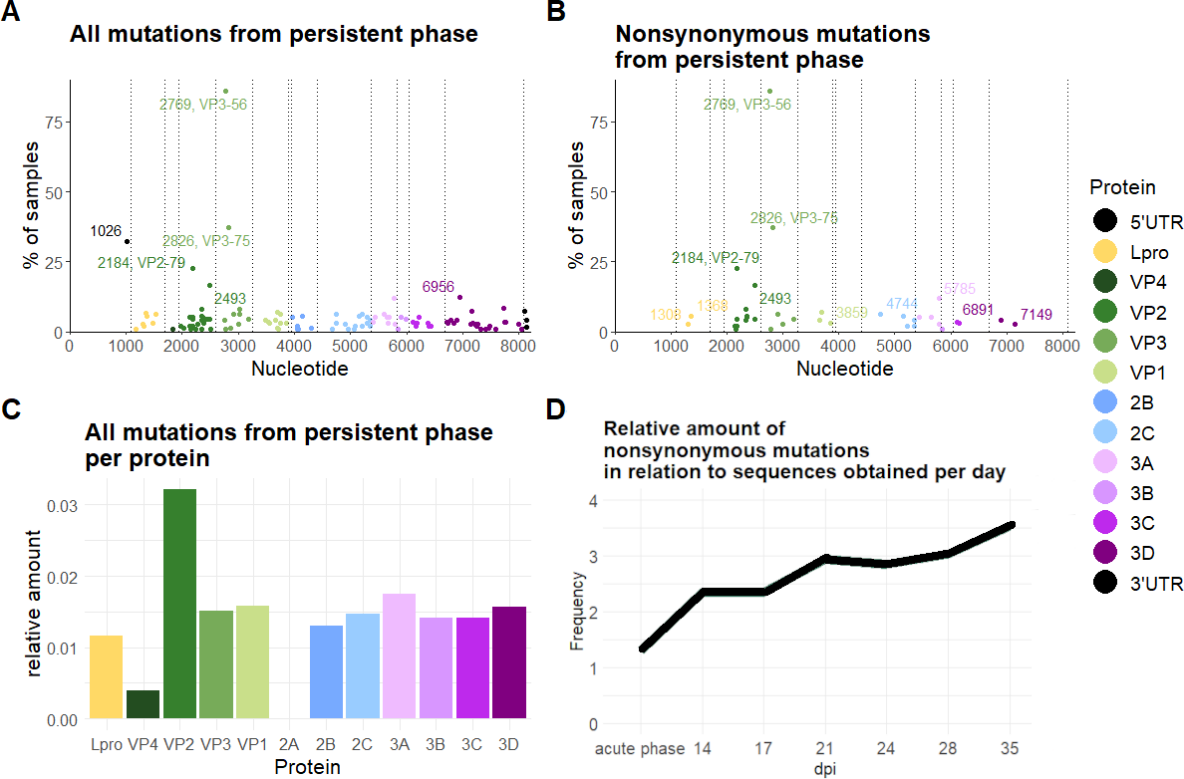
Persistent-phase mutations. Mutations detected in the transitional and persistent-phase OPF samples. Mutations are shown with their position in the genome and the occurrence of each mutation in percent of all samples, for all mutations (**A**) or only non-synonymous mutations (**B**). The affected amino acid is annotated for the three most frequent non-synonymous mutations. The counts of all non-synonymous mutations were summarized by virus protein, and the amount of mutations was divided by the length of the protein (**C**). The amount of mutations per day was divided by the number of sequences obtained for each day to calculate the relative occurrence of mutations (D).

In the consensus sequences (*n* = 97) recovered from the OPF in the transitional and persistent phases, 142 mutations were detected. Thereof, 46 mutations coded for an amino acid change. A detailed list of non-synonymous nucleotide mutations by day and animal is shown in Fig. 5. The mutations that were present in most samples are three non-synonymous mutations VP3 R56C (found in 84 samples), VP3 A75T (in 36 samples), and VP2 Y79H (in 22 samples), together with a silent mutation at nucleotide 1026 in the 5′ UTR (in 28 samples) as shown in Fig. 6A and B. After grouping the mutations by protein, the highest number of mutations was detected in the polymerase 3D with 22 mutations, followed by VP2 with 21 mutations. Divided by the length of each coding region, the largest protein of FMDV 3D has a low frequency of mutations, while VP2 still has the highest number of mutations relative to the length of its coding region (not shown). This is further emphasized when only non-synonymous mutations are considered: the capsid proteins VP2 and VP3 had the most non-synonymous mutations, in absolute numbers (not shown) and relative to the length of their coding regions (Fig. 6C).

### Mutations over time

To quantify the occurrence of non-synonymous mutations over time, all samples with mutations compared with the inoculum were counted for each sampling day following 14 dpi. All mutations found in samples from the acute phase were grouped together irrespective of the sampling day. In absolute terms, the frequency of non-synonymous mutations declined after the acute phase to reach the lowest point on day 14 with 22 non-synonymous mutations found in samples collected on this day. Relative to the number of whole-ORF consensus sequences recovered on each day, a steady increase in the frequency of mutations was observed over the course of infection, reaching a peak at 35 dpi with a frequency of 3.5 (Fig. 5D), meaning that on average, in every sequence recovered from this day, more than three mutations were found relative to the inoculum.

**Fig 7:**
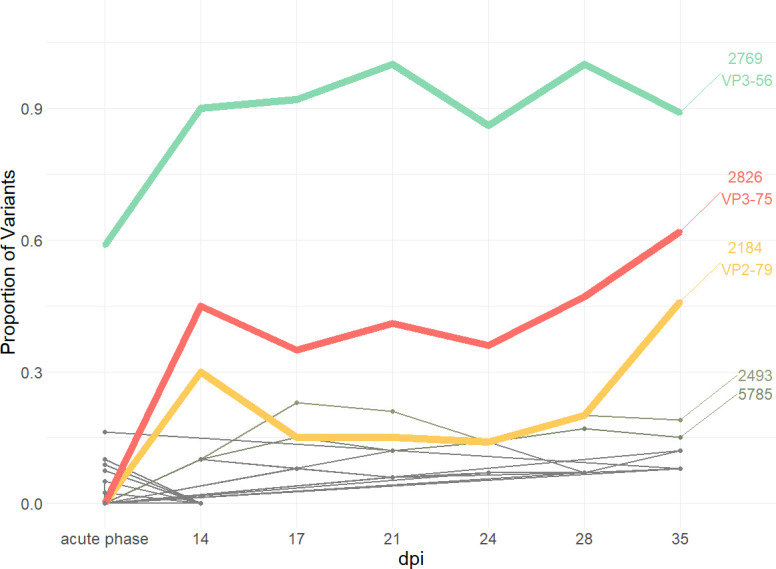
Non-synonymous mutations by sampling day. Observed frequency of specific non-synonymous mutations over time during the acute phase (3–6 dpi) and on each sampling day in the transitional and persistent phases.

There were distinct sets of mutations occurring only during the acute phase or during the transitional and persistent phase. Only a few mutations that were already present in sequences from the acute phase remained detectable in the consensus sequences recovered from OPF samples (including VP3 R56C and VP3 G60D), while the majority of non-synonymous mutations disappeared after the acute phase. Interestingly, we saw an increase in the emergence of non-synonymous mutations over time at nucleotides 2826 and 2184, coding for amino acid exchanges in VP3 (A75T) and VP2 (Y79H) (Fig. 7A). While the non-synonymous mutation at position 2769 coding for VP3 R56C was already present in more than half of the samples collected during the acute phase and was subsequently found in almost all OPF samples, VP3 A75T and VP2 Y79H were not detected in any consensus sequence from the acute phase nor as a minor variant in the deep-sequenced vesicular samples. They were also not present as minor variants in the deep-sequenced inoculum but appear in the consensus sequences from 14 dpi onward, with VP3 A75T present in 41% and VP2 Y79H in 27% of samples from this day. At 35 dpi, VP3 A75T occurs in more than half of the samples (53%) and VP2 Y79H almost in every second sample (47%). They were found in several animals regardless of the vaccination group, and VP2 Y79H occurred independently in both animal trials. VP3 A75T was detected at the consensus level in eight animals from the vaccination trial (ear tags 190, 191, 196, 425, 507, 509, 647, and 669) but was not found in the two persistently infected animals from the infection study. In contrast, VP2 Y79H was present in five cattle from the vaccination study at the consensus level (ear tag numbers 507, 510, 649, 943, and 945) as well as in one animal from the infection study (773). Additionally, VP2 Y79H was observed as a heterozygous base in the Sanger consensus sequence of four animals (191, 196, 506, and 507), adding up to 10 of 20 persistently infected animals in this study in which this non-synonymous mutation was found.

### Structural analysis

**Fig 8:**
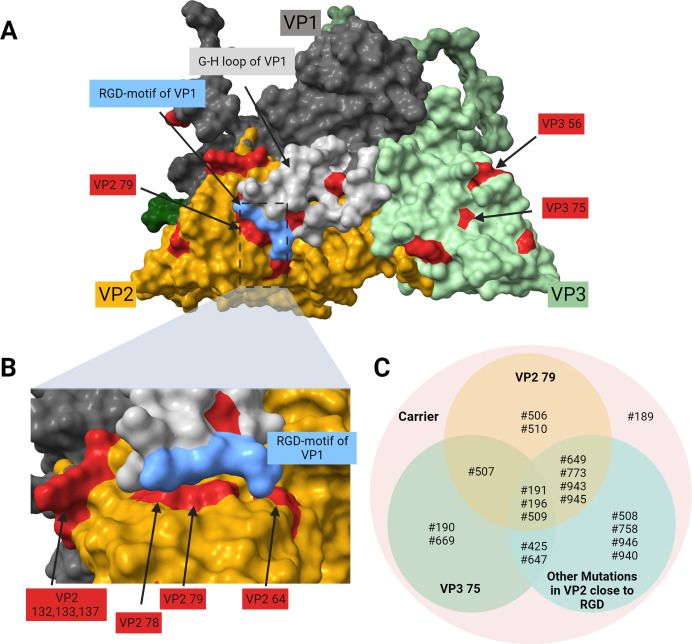
Localization of amino acid changes in the virus capsid. (**A**) Amino acid changes (colored red) on the capsid surface; the G-H loop of VP1 in the “down” position is colored in light gray, including the RGD residues 145–147 shown in blue. (**B**) Detailed view of the vicinity of the G-H loop with the RGD motif in the “down” position, with the amino acid changes in VP2 again shown in red. (**C**) Venn diagram of persistently infected animals in which certain amino acid changes on the capsid surface were observed; “other mutations in VP2 close to RGD” include the residues at positions 64, 78, 79, 132, 133, 137, and 182.

The localization of the non-synonymous mutations in the capsid proteins was hypothesized to have functional implications. After mapping the amino acid changes on the capsid, the first observation was that the majority of amino acid changes lies on the outer surface of the capsid (Fig. 8A) and none on the inner surface. Important structures on the surface are the five major antigenic sites and the receptor-binding sites.

The following amino acid changes have been observed to constitute immunologically relevant major antigenic sites or lie in close proximity to these: VP1 residue 144, VP2 residues 64, 78, 79, 132, 133, 137, and 182, and VP3 residue 56 (6). VP1 144 is a central amino acid of the antigenic site 1 in the GH loop (25). The mentioned amino acids of VP2 (except 182) lie close to the “antigenic site 2” on VP2 (26). The exchanged amino acid VP2 182 (found in six animals) is not exposed on the surface in our AlphaFold prediction of VP2 of O/FRA/1/2001 but lies in the β-strand H directly beneath the amino acid residue 79 located in β-strand C. VP3 56 is a critical residue of the “antigenic site 4,” enabling escape mutants (27).

Using an X-ray crystallography model in the “down” position, we observed an accumulation of amino acid changes on the surface of VP2 in direct vicinity of the RGD motif of the GH loop of VP1. In addition to forming the “antigenic site 2,” VP2 residues 64, 78, and 79 are located right beneath the RGD motif (Fig. 7B). The motility of the GH loop allows a change between an “up” and a “down” constitution, simultaneously altering its own antigenicity (7, 28).

An amino acid change in residue VP3 79, which lies outside the canonical antigenic sites, was previously linked with mutation pressure in the presence of antibody (29).

Apart from the antigenic sites, some residues constitute receptor-binding sites as well, such as VP3 56 and VP2 residues 78, 79, 132, and 133, which form the canonical HS-binding sites. Arginine at VP3 56 can interact with sulfide groups on heparin, while the other residues support interactions through local charges (30, 31). A detailed description of which mutation occurred in what animals is shown in Fig. 7C.

Furthermore, both amino acid changes that were observed to emerge during FMDV persistence, VP3 A75T and VP2 Y79H, are located in regions associated with T-cell epitopes (32).

### Viral dynamics within one animal

**Fig 9:**
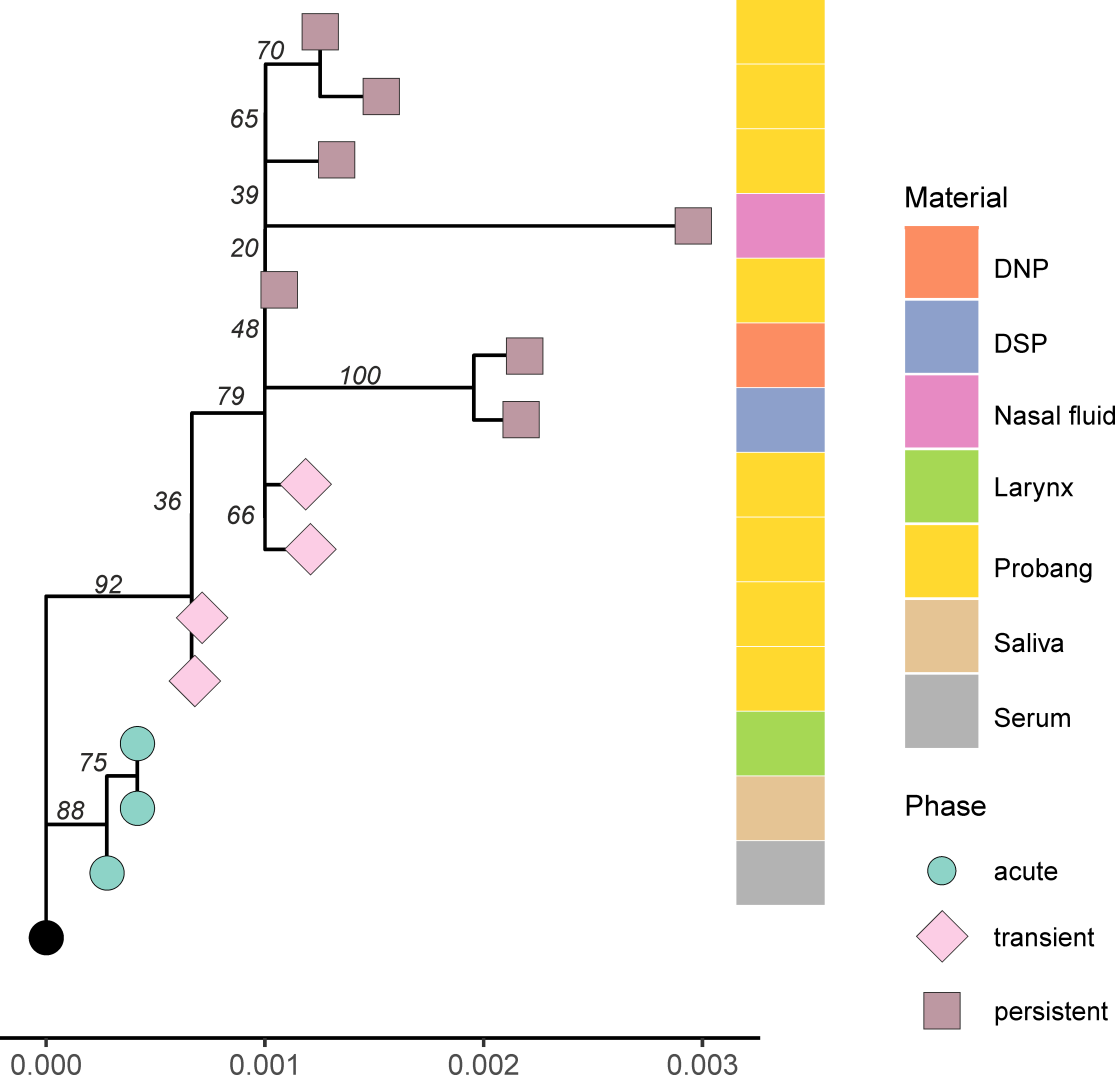
Inoculum-rooted phylogenetic analysis of all sequences recovered from animal 758 categorized by phase of infection and sampling material. Samples from the acute phase were taken from 3 to 6 dpi, from the transient phase from 10 to 21 dpi, and from the persistent phase after 21 dpi; tissue samples including DNP, DSP, and larynx epithelium were collected during necropsy at 35 dpi.

The diversity of the viral population within one animal was examined using different samples collected from animal 758. We used three samples from the acute phase (serum, nasal fluid, and saliva), eight OPF samples, and three tissue samples collected at necropsy at 35 dpi (dorsal soft palate, dorsal nasopharynx, and larynx epithelium from the bottom of the epiglottis; all were positive in the virus isolation). The tissue samples are the only samples included in this study in which sequencing was performed after one passage on LFBK-αVβ6. The tissues used here are also the preferential localization for persistent FMDV (33). In samples from the acute phase, we detected two to three mutations in each consensus sequence. During the persistent phase, this number rose from two mutations in OPF from 10 dpi to six mutations at the consensus level in OPF samples from 28 and 31 dpi. In the virus isolates from the tissue samples from the end of the trial, 10 to 17 mutations were found. The consensus sequences from the DSP and DNP were highly similar with nine shared mutations compared with the inoculum, while the larynx sample shared only 3 of its 17 mutations with the other tissues. In a phylogenetic analysis, DNP and DSP samples were closely related to each other and to the OPF samples, while the sequence from the larynx sample had no close relation with any other sample from the persistent phase (Fig. 9). The larynx sample is the only sample from this animal in which the amino acid change VP2 Y79H was detected.

One non-synonymous mutation shared by all OPF samples and tissues codes for an amino acid change in VP2 (D133G), which is located on the capsid surface. Interestingly, animal 758 is also the only animal in both trials in which the VP3 R56C variant was dominant during the acute phase but disappeared from the consensus sequences in OPF samples. Overall, we observed a disparity between the high amount of mutations found in persistently infected epithelia in the nasopharynx and the reduced presence of mutations in the consensus sequences of OPF samples.

### Functional analyses

The selection of isolates that met our requirements for a functional analysis was confounded by the diversity of isolates obtained by plaque purification. This broad diversity of sequences from the plaques reflects the nature of the viral quasispecies in the persistently infected animals. VP3 R56C was fixed in all sampled plaques. Several plaques shared mutations, among them amino acid changes, which were also observed in other OPF isolates, such as VP1 144 (found in 5 of 25 plaques of the isolate obtained from animal 510 at 14 dpi) and VP2 182 (found in 12 of 27 plaques of the 508/24 dpi isolate; this variant becomes the consensus of this animal at 28 dpi). We selected plaque-purified isolates corresponding to the consensus sequence of the inoculum (plaque #2) and two isolates from the persistent phase, one with the VP3 R56C mutation (plaque #65) and one with VP3 R56C and VP2 Y79H (plaque #57). These mutations were the only non-synonymous mutations in the P1 of both isolates.

**Fig 10:**
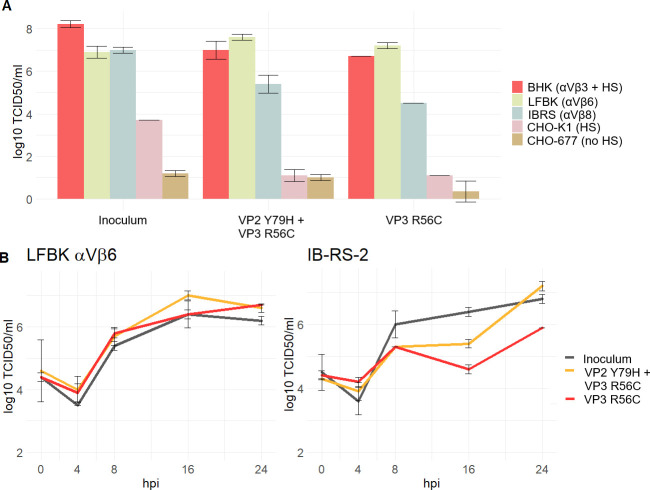
Characterization of the plaque isolates. Plaque identical to the inoculum consensus, plaque with VP2 Y79H and VP3 R56C, and plaque containing VP3 R56C only. (**A**) Infectivity assay with receptor-deficient cell lines; the main receptors are indicated in the brackets: integrins αVβ3, αVβ6 or αVβ8, and HS. (**B**) Comparison of viral growth kinetics of the plaque isolates on LFBK-αVβ6 and IB-RS-2 cells over 24 hpi.

To test the ability of each variant to infect different receptor-deficient cells, the isolates were used to infect five cell lines. All isolates were able to productively infect integrin-expressing cells (BHK-21, LFBK-αVβ6, and IB-RS-2) and reached high titers. In integrin-deficient but HS-expressing CHO-K1 cells, only the plaque isolate with the inoculum sequence (#2) was able to reproduce, albeit at decreased titers. In contrast to this, no isolate could productively infect the integrin- and HS-deficient cell line pgsD-677. The isolates from the persistent phase were unable to infect cells using HS alone (Fig. 10A).

The differences between the two integrin-expressing cell lines were further evaluated using growth kinetics on LFBK-αVβ6 and IB-RS-2 cells. In the LFBK-αVβ6 cells, which express the bovine αVβ6 integrin (34), all three isolates showed very similar growth with slightly lower titers for the inoculum isolate. In IB-RS-2 cells, which express αVβ8 integrin but not αVβ6 (35), the inoculum isolate grew unimpeded, similar to LFBK-αVβ6 cells. Both isolates of persistent viruses seem to exhibit retarded growth but not to a statistically significant extent (Fig. 10B).

In the virus neutralization assay, we observed no difference in the neutralizing activity of serum collected from persistently infected animals (ear tags 508 and 510) at 35 dpi against the plaque-purified inoculum and the isolates from persistently infected animals (data not shown).

## DISCUSSION

Understanding the replication of FMDV in the acutely and persistently infected host is of great importance. Heterologous superinfection of persistently infected cattle gives rise to inter-serotype recombinants that can evade the host immune response by replacing the capsid-coding region of their genome (36, 37). However, in addition to this sudden antigenic shift caused by recombination between serotypes, FMDV continuously evolves over the course of infection in persistently infected carriers. What we observed in this study constitutes a genetic drift with a steadily increasing amount of mutations accumulating in the viral genome. In addition to this accumulation of mutations, another piece of evidence for the ongoing viral replication in the persistent phase is the presence of negative-strand viral RNA in tonsils and DSP of persistently infected sheep (38).

We avoided culture passages of virus recovered from the animals prior to sequencing to avoid culture-induced mutations. Sequencing FMDV directly from the *ex vivo* samples may have reduced our sample set slightly but supplied sequences as close to the natural infection as possible. The importance of this approach is emphasized by a comparison with the same samples after one culture passage, which revealed different consensus-level mutations in several corresponding sequences. Although many mutations were preserved through a cell culture passage, even a single passage can introduce many unique mutations, which seem to be randomly distributed over the genome. However, amplification of viral RNA through PCR may alter the relative frequencies of quasispecies variants, potentially skewing the consensus sequence away from the true population diversity.

Our phylogenetic analysis has shown that in the same animal, many samples are highly homologous with only single mutations occurring between sampling timepoints, but single clade-shifted samples were found to cluster with samples from a different animal rather than with others from the same animal. Similar patterns of phylogenetic relation have been observed before (19). The original hypothesis of Fish et al., (20) that this presence of viral quasispecies within the same animal can be explained by a multitude of infection foci with independent replication, is supported further by our finding of divergent sequences of FMDV in different tissues collected from the same animal at necropsy. Even in our limited sample set of virus-isolation positive tissues of animal 758, a distinct set of viral subpopulations was present in each of the tissues. The subpopulations in DSP and DNP differed only in a few nucleotides, which may be associated with their close anatomic localization, while FMDV recovered from laryngeal epithelium was clearly different from the two others with several mutations that were not detected in the OPF of animal 758 but were present in the consensus sequence of OPF from other animals, including amino acid change VP2 Y79H. The disparity between the number of mutations observed in tissue isolates and in OPF might not be fully attributable to the cell culture passage, as many of the single nucleotide polymorphisms (SNPs) were shared between DNP and DSP tissue isolates. Vice versa, the consensus sequence of the OPF of this animal mostly contained mutations that were found in the tissues as well. This suggests that OPF encompasses a broad variety of subspecies, and its sequence is drawn from a set of individual subpopulations. This may be explained by the technique of probang sampling to collect OPF. The metal probang cup is inserted through the mouth and swallowed by the animal, then saliva and superficial scrapes of epithelium are collected by retracting the cup (8, 39). Therefore, the probang cup samples viral subpopulations from this entire region, but it will not always be exactly the same foci of infection that are sampled on different days.

Another interesting finding of this study was the emergence of three non-synonymous mutations, coming to dominate the consensus sequence in several animals. Among these, the non-synonymous mutation coding for VP3 R56C was the variant with the highest prevalence across samples, being detected in over 90% of all samples from the persistent phase. However, this variant was already present in the inoculum at a frequency of 22% and already constituted the consensus in over half of the samples from the acute phase (3–6 dpi). It was found in all persistently infected animals except animal 758, from which it disappeared after the acute phase. Residue 56 of VP3 is well characterized, and arginine at this position is associated with attenuation *in vivo* and increased HS binding (40). Especially the substitution of arginine (R) with cysteine (C) was associated with a reversion to pathogenicity (21) and was quickly introduced by serial passages in cattle (41). Remarkably, most of the field strains for which sequence information is available in online databases have a histidine at this position (93% of 479 sequences), in contrast to only very few sequences with a cysteine at residue 56, which all come from the aforementioned passage experiment. Juleff et al. (41) used intradermolingual inoculation of cattle to obtain vesicular material for further passage, like we did for our inoculum. In our study, this mutation was likely induced by the animal passage of the previously plaque-purified inoculum before the infection of animals in the vaccination trial, as a comparison with closely related field strains from the 2001 outbreak implies as well. The inoculum of the infection trial, on the other hand, does not have a history of animal passage, and the emergence of VP3 R56C was associated only with the acute phase. After the acute phase, it was no longer detected at the consensus level in animal 758. In animal 507, arginine similarly reappears at residue 56 on days 24 and 35. The dynamic of the occurrence and shortly thereafter the disappearance of VP3 R56C in these animals, as well as the distinct mutations acquired during the acute and the persistent phases, may reflect the viral subpopulation that initially infects the FAE of the nasopharynx then spreads from this microanatomic compartment throughout the body, where it acquires several mutations during rapid replication and is cleared with the onset of the antibody response. In the FAE, however, the viral subpopulation from the primary infection may remain and replicate slowly but steadily, thus “conserving” its genetic makeup.

In contrast to the VP3 R56C variant, which is tied to the acute phase of infection, two other non-synonymous mutations were associated with the persistent phase: VP2 Y79H and VP3 A75T. These were detectable neither in the deep-sequenced inoculum or vesicular lesions nor in any consensus sequence of other samples from the acute phase. They first appear in our samples at 14 dpi, increasing in prevalence toward the end of the trial, whereupon they appear in the consensus of half of the samples. Especially for VP2 Y79H, there is an astonishing presence of ambiguous base calls. VP2 Y79H has previously been found to be associated with FMDV persistence in an animal trial with FMDV O UKG/34/2001 (a very close relative of O FRA/1/2001), where it was present in six of six carrier animals (16). In another experimental study with FMDV O UKG/34/2001, VP2 Y79H was detected in four of six samples from three carrier animals, while in the same study, VP3 A75T was found in five of the sequences (18). Parthiban et al. (18) did not assert an association of these samples with persistent infection because the same substitutions were also found in samples collected from acutely infected animals in the field. In a comparison of 479 FMDV serotype O sequences available in online databases, histidine is indeed the dominant amino acid at residue 79 of VP2 (occurring in 70.5% of samples). However, in our view, this does not necessarily contradict the association of VP2 Y79H with the persistence of FMDV O/ME-SA/PanAsia (i.e., O FRA and O UKG 2001), because most of the field viruses in the database are not of this topotype. In a comparison of PanAsia strains, only an isolate of O/JPN/2000 that had been obtained from OPF (42) had a histidine at residue 79 (43). What is more, in the FMD epizootic in the United Kingdom in 2001, where the transmission of O UKG between acutely infected animals took place over the course of nearly 7 months, VP2 Y97H was not observed in any of the field samples taken over the whole period, and neither was VP3 A75T (23). The commonness of histidine at residue 79 of VP2 in other strains, however, may limit our findings to PanAsia strains. While similar associations with FMDV persistence could be assumed for neighboring residues of VP2 79 on the capsid surface, these would require a more detailed analysis.

For VP3 residue 75, an analysis of the publicly available sequences of FMDV serotype O (*n* = 479) revealed threonine to be the least abundant amino acid at this position with an occurrence of 14%, while alanine was dominant with 84%. In an infection experiment with O_1_ Manisa in cattle, VP3 A75T was found in samples taken from one persistently infected animal but only after the 21st day after infection (accession numbers MT431591–MT431613) (36, 44). In general, information about this amino acid is scarce, but it could potentially be associated with FMDV persistence as well.

We observed no antigenic differences when testing the neutralizing capacity of serum from the persistent phase against the inoculum and a late isolate from a carrier animal. This is in agreement with the findings of the previous study of VP2 Y79H (16) and with neutralization tests performed with serum and virus recovered from persistently infected African buffalo (45). Testing the neutralizing capacity of monoclonal antibodies against our plaque-purified isolates would allow a more detailed study of their antigenic character, especially considering the antigenic site 1 in the G-H loop, whose antigenicity may be influenced by the closely located amino acid changes on the surface of VP2. The accumulation of amino acid changes in critical residues of antigenic sites and receptor-binding motifs suggests a fitness advantage conferred by these changes during viral persistence. Gebauer et al. found differences in the neutralizing capacity of two of her persistent-phase isolates with monoclonal antibodies (15), while Salt could not corroborate this in his experiments (46). However, the possibility that the mutations influence the T-cell epitope should not be disregarded, but testing this hypothesis lies outside of the scope of this study.

We further investigated the functional differences between VP2 Y79H and VP3 R56C. We chose an isolate with both mutations, VP2 Y79H and VP3 R56C, because the variant VP2 Y79H was detected independently in sequences from both trials, but no plaque isolate had VP2 Y79H as the only amino acid change without VP3 R56C. As suggested by previous studies of the VP3 R56C mutation, both persistent isolates (one containing VP2 Y79H and VP3 R56C, and one containing only VP3 R56C) lost their ability to infect integrin-deficient but HS-expressing cells. This may demonstrate the evolution of animal-passaged viruses away from the cell-culture-adapted original isolate; however, using hamster-derived cells is quite artificial, and the growth of certain virus isolates may be inhibited by unknown other factors in these cell lines. Based on the growth kinetics, VP2 Y79H may pose an advantage over VP3 R56C alone by enabling an infection of cells via the integrin receptor αVβ8, but in our case, this trend was not significant and may also be influenced by other factors. This receptor binding is facilitated as well by the RGD motif in VP1 (47) and would thus be influenced by VP2 79, which is in close vicinity to the G-H loop in its “down” position. In contrast to αVβ6, the integrin receptor αVβ8 is not only expressed on epithelial cells, but also on T_reg_ cells and dendritic cells, which are present in the FAE (48, 49), and at least dendritic cells can be infected by FMDV under certain circumstances (50).

Our results show that in FMDV serotype O infections, there are indeed unique mutations associated with the persistent phase of infection. In addition, the VP2 Y79H variant may confer a functional change in receptor usage. However, the broader implications of both findings currently remain unclear.

## MATERIALS AND METHODS

### Cells

BHK-21 cells (Collection of Cell Lines in Veterinary Medicine [CCLV]-RIE 164, FLI, Greifswald, Germany) and IB-RS-2 (CCLV-RIE 103) (51) were cultured in minimum essential medium (MEM) with Hanks’ and Earle’s salts and non-essential amino acids. LFBK-αVβ6 (CCLV-RIE 1419) (34), CHO-K1 (CCLV-RIE 134) (52) and CHO-677 cells (pgsD-677, CCLV-RIE 1524) (53) were cultured in medium containing Ham’s F12 Nutrient Mixture and Iscove’s modified Dulbecco’s medium (IMDM) with sodium bicarbonate. For routine propagation, 10% fetal bovine serum (FBS) was added.

### Viruses

All experiments were performed with FMDV O/FRA/1/2001. For the two animal trials, two differently prepared inocula with the same consensus sequence (GenBank accession no. OV121130.1) were used. For the first trial, a vaccination trial, virus was provided by the Animal Health Laboratory, ANSES, Maisons-Alfort, France. It had been plaque-purified twice on BHK-21 cells and was then passaged once in a heifer that was infected by intraepidermolingual inoculation. The inoculum was prepared from a vesicular lesion on the tongue of this animal collected at 1 dpi. For the second trial, an infection study, the inoculum was recombinantly produced O/FRA/1/2001 and passaged twice on BHK-21 cells.

### Animal trial

The samples used in this study were collected in two separate animal trials. Both were carried out under BSL4vet conditions at the Riems site of the Friedrich-Loeffler-Institut (Germany). The majority of samples stem from a vaccination trial in which 20 Holstein-Frisian cattle (*Bos taurus*) were challenged by intranasopharyngeal instillation with the animal-passaged inoculum. Prior to challenge infection, six animals had been vaccinated twice intramuscularly (i.m.) with a commercially available inactivated FMDV O_1_ Manisa vaccine, six animals had been vaccinated three times intranasally with an experimental canine adenovirus vector vaccine expressing P1 and 3C of FMDV O/FRA/1/2001, four animals had been vaccinated three times i.m. with the same vector vaccine, and one control group of four animals had been injected with sterile phosphate-buffered saline (PBS). All animals remained in the trial until the 35th day after the challenge, at which point they were euthanized.

The samples from animals 758 and 773 were obtained from an infection experiment previously described in Litz et al. (54). Here, the intranasopharyngeal inoculation was performed with recombinant O/FRA/1/2001, which had the same consensus sequence as the inoculum of the experiment described above. All animals remained in the trial until 35 dpi.

### Sample collection

Serum, nasal fluid, and saliva were collected daily from 0 to 10 dpi and on days 14, 17, 21, 24, 28, 31, and 35 postinfection. Serum was collected from the jugular vein into tubes containing clotting activators. Saliva samples and nasal fluid were obtained by swabbing the oral or nasal cavity with a human vaginal tampon as previously described (55). Probang samples were collected on days 0, 14, 17, 21, 24, 28, and 35. In the infection study, additional probang samples were performed at 10 and 31 dpi, as described by Sutmoller and Cottral (13). In the infection study, tissue samples from the nasopharynx were collected at the time of necropsy. To address the focal nature of persistent FMDV, five biological replicates of each of the preferred epithelia, the dorsal nasopharynx, and the dorsal soft palate, and one sample of epithelium at the base of the epiglottis were taken from each animal. These tissue samples were used for viral genome detection and virus isolation. All samples were frozen at −80°C on the day of collection pending further analysis.

### Virus isolation from probang samples

To determine the carrier status of FMDV-infected cattle, virus isolation was attempted from the OPF collected with the probang cup (13). An equal amount of approximately 4 mL of cell culture medium was added to the sample and then it was homogenized through repeated aspiration using a 1.7 mm blunt cannula. The homogenate was aliquoted, and one part was stored at −80°C untreated while approximately 2 mL was mixed with an equal amount of 1,1,2-trichloro-1,2,2-trifluoroethane (TTE) (56). The OPF with TTE was then vigorously shaken for 5 minutes and centrifuged at 1,000 × *g* at 4°C for 10 minutes. The supernatant was removed and aliquoted for virus isolation, which was performed with 250 µL of the TTE-treated OPF on a confluent monolayer of LFBK-αVβ6 cells.

### FMDV genome detection

For FMDV genome detection, RNA was extracted from 100 µL of serum, nasal fluid, saliva, and untreated OPF using the NucleoMag Vet kit (Macherey-Nagel) and a King Fisher Flex magnetic particle processor (Thermo Scientific). As an internal control, 10 µL of IC2 RNA was added to each sample (57). An RT-qPCR using AgPath-ID One-Step RT-PCR reagents (Thermo Fisher Scientific) with a primer/probe set targeting the 3D-coding region (58) was performed to detect FMDV RNA.

### RNA extraction and sequencing

For the sequencing of FMDV genomes, the untreated OPF, as well as samples of serum, nasal fluid, and saliva collected during the acute phase, was used. RNA extraction was performed with 250 µL of the original sample (without prior amplification of virus in culture) and 750 µL TRIzol LS Reagent (Thermo Fisher Scientific) as described by the manufacturer. To obtain a sequence of the entire ORF, a set of 10 primer pairs was chosen, each spanning 900 to 1,000 nucleotides with overlapping ends, as described in Table S3. Apart from the FMD Mix 3 of Dill et al. (59), all primer pairs were specifically designed for FMDV O/FRA/1/2001 using Primer3web version 4.1.0 (https://primer3.ut.ee/) (60). The genome was amplified using the qScript XLT One-Step RT-qPCR ToughMix (Quantabio) on a SimpliAmp Thermocycler (Thermo Fisher Scientific) with the following temperature profile: 20 minutes at 48°C for reverse transcription, 3 minutes of activation at 94°C, 45 cycles of 15 seconds at 94°C, 30 seconds at 60°C, 60 seconds at 68°C, and a final elongation of 5 minutes at 68°C. Afterward, the DNA amplicon was purified by gel electrophoresis. Bands of the expected lengths were excised, and a clean-up was performed with the QIAquick Gel Extraction Kit (Qiagen). The purified DNA was sent to Eurofins Genomics (Ebersberg, Germany) for Sanger sequencing.

For comparison, supernatants of virus isolations from the same OPF samples on LFBK-αVβ6 cells were partially sequenced using the primer sets of Dill et al. (59), covering the region coding for the polyprotein P1 and a region spanning from 2C to 3B.

A selected set of samples was used for deep sequencing, including the homogenized vesicular material used as the inoculum of the vaccination trial and vesicular fluid collected from the interdigital cleft of six animals during the acute phase (animals 190, 191, 425, 508, 509, and 662). Total RNA was extracted, converted into cDNA libraries, and sequenced as previously described (61). In short, RNA was extracted with TRIzol LS (Thermo Fisher Scientific) and RNeasy Mini spin columns (Qiagen) with on-column DNase I digestion. Subsequently, 500 ng of total RNA was reverse transcribed using SuperScript IV First-Strand cDNA Synthesis System (Thermo Fisher Scientific) in combination with the NEBNext Ultra II Non-Directional RNA Second Strand Synthesis Module (New England Biolabs), according to the manufacturers’ instructions. The cDNA was sheared on a Covaris M220 Focused-ultrasonicator with a target size of 400 bp. Sheared DNA was concentrated by adding 1.8 volumes Agencourt AMPure XP beads (Beckman Coulter), washed twice with 80% (vol/vol) ethanol, eluted in 25 µL nuclease-free water, and used for library preparation with the GeneRead DNA Library L Core kit (Qiagen). After end repair and adapter ligation, the library was purified using 1.8 volumes AMPure XP beads, followed by a size selection aiming at a target size of around 350 to 550 bp. Quality control was performed with an Agilent High Sensitivity DNA kit, and the molarity was determined with a KAPA Library Quantification kit. Sequencing was performed in a pooled run on an Ion Torrent S5 XL instrument with an Ion 530 Chip Kit.

### Sequence analysis

Sanger sequencing reads were mapped to the consensus sequence of the inoculum, and a consensus sequence of the ORF of each sample was generated with Geneious Prime 2021.0.1 (https://www.geneious.com). Sanger reads with low-quality chromatograms (e.g., high background or no discernable peaks) were excluded. For most of the ORF, this consensus sequence was created from two overlapping, quality- and primer-trimmed Sanger reads of 900–1,000 nucleotides in length. In Geneious Prime, the consensus threshold was set at 75% and heterozygotes were called at 50%. The “Find Variations/SNPs” tool in Geneious was used to detect discrepancies between the mapped sequences and the consensus sequence of the inoculum.

For deep-sequenced samples, data sets of 1.6 to 4.4 million reads were quality trimmed with Trimmomatic version 0.39 (https://github.com/usadellab/Trimmomatic) using an average quality of 22 in a sliding window of 5-mers. Additionally, reads shorter than 50 bp were discarded. In general, during quality trimming, around one-third of the input reads was dropped, leaving two-thirds of the data set as high-quality data for further analysis. The trimmed reads were mapped against the full genome sequence of the twice plaque-purified cell culture isolate (GenBank accession no. OV121130) using Bowtie2 (https://github.com/BenLangmead/bowtie2; version 2.3.4.3) in the --sensitive mode (end-to-end alignment), with 34% of the data set aligned (inoculum) or >95% (vesicular fluid), respectively. Variants were called using LoFreq (https://github.com/CSB5/lofreq; version 2.1.3.1) with default filter settings. Variants with <0.5% frequency were excluded from further analysis and interpretation. Consensus-level mutations were defined as variants that appeared in more than 50% of the reads with respect to the original inoculum.

Detailed analysis and graphical representation of mutations were performed using R (https://www.r-project.org/). Visualization of mutations in the capsid was conducted with ChimeraX (62, 63) based on the X-ray crystal structure of 1FOD, an FMDV O1K strain including the G-H loop in a “down” position (7). The VP2 capsid protein structure of O/FRA/1/2001 was predicted using AlphaFold (64) in ChimeraX.

### Phylogenetic analysis

An alignment of all sequences including the inoculum was prepared using MUSCLE (version 3.8.425 [65]) in Geneious. From this alignment, a distance matrix based on the differences in the alignment was constructed in R (https://www.r-project.org/) and calculated using classical multidimensional scaling (66). A phylogenetic tree was constructed with IQ-TREE2 (version 2.3.6) using automatic model selection and 10,000 ultrafast bootstrap replicates.

### Functional analyses

Functional analyses were performed comparing the inoculum and two isolates from the persistent phase of infection, one containing only one amino acid exchange in VP3 (R56C) and one isolate containing VP3 R56C as well as an amino acid exchange in VP2 (Y79H).

#### Plaque purification

To obtain virus isolates with the appropriate genotypes, plaque purification was performed with the inoculum, TTE-treated OPF from animal 508 collected at 24 dpi, and TTE-treated OPF from animal 510 collected at 14 dpi, which was passaged once on LFBK-αVβ6 cells. The plaque purification was carried out on a 90% confluent LFBK-αVβ6 monolayer with an overlay of 1.5% methyl cellulose (Sigma-Aldrich) in cell culture medium. Plaques were picked after 24 to 48 hours with a pipette tip, transferred to culture medium, incubated overnight, and passaged once on LFBK-αVβ6 cells until a cytopathic effect was apparent. The desired mutations were confirmed by sequencing the P1-coding region with the primer pairs described above.

#### Virus titration

Endpoint titration was conducted on LFBK-αVβ6 cells. Viral titers were calculated as 50% tissue culture infectious doses (TCID_50_) per 100 µL using the Spearman-Kärber method (67, 68).

#### Infectivity assay on receptor-deficient cells

The infectivity assay was adapted from Jackson et al. (69), without washing the cell monolayers with citric acid buffer. The receptor-binding capacity of the plaque-purified viruses was evaluated by infecting cells expressing different receptors FMDV can utilize for cell entry. This included LFBK-αVβ6 cells expressing the preferred integrin receptor αVβ6; IB-RS-2 cells expressing only integrin αVβ8 but not αVβ6 (35); BHK-21 cells expressing the integrin αVβ3 and HS, but for efficient FMDV infection, HS is required (70); CHO-K1 cells expressing only HS but no integrins (71); and CHO-677 cells expressing neither integrins nor HS (53). These experiments were performed in duplicate. Confluent monolayers of cells were infected, incubated for 1 hour at 37°C, and then washed with culture medium. Supernatant from the cultures was harvested 16 hours postinfection.

#### Growth kinetics

Viral growth on LFBK-αVβ6 and IB-RS-2 cells was characterized and compared between the three isolates. Cell monolayers in 12-well plates were infected at a multiplicity of infection of 0.1. Samples were taken at 0, 4, 8, 16, and 24 hours postinfection and were titrated on LFBK-αVβ6 cells (72). These experiments were performed in duplicate.

#### Virus neutralization test (VNT)

A neutralization test to compare the inoculum and plaque-purified virus from the persistent phase was performed using the serum collected from the same animal. The VNT was performed as described by the World Organisation for Animal Health (WOAH) (73). Briefly, a twofold serial dilution of serum was prepared in a 96-well plate in duplicate. To each well, 50 µL of virus suspension containing 100 TCID_50_ was added. After an incubation of 2 hours at 37°C, 50 µL of a BHK-21 cell suspension was added. The plates were read after 3 days at 37°C with 5% CO_2_. Titers are expressed as the final dilution of serum where 50% of wells were protected from cytopathic effect.

### Statistical analysis

The binomial proportion confidence interval for the incidence of persistent FMDV infection was calculated by the Wilson method (74) using R (https://www.r-project.org/).

## Data Availability

The deep sequencing data used in this study were uploaded to SRA under accession no. SRR31675832–SRR31675838. The consensus-level sequences spanning the FMDV ORF from 21 animals along with metadata were published in Zenodo project 10.5281/zenodo.14384049.

## SUPPLEMENTAL MATERIAL

Figure S1 jvi.01422-24-s0001.docx Body temperature and vesicular lesions of the animals in the vaccination study.

Table S1 jvi.01422-24-s0002.xlsx Detected single nucleotide variants (SNVs) in inoculum and vesicular material from different animals.

Table S2 jvi.01422-24-s0003.xlsx Non-synonymous consensus-level amino acid variants detected during the acute phase of FMDV infection.

Table S3 jvi.01422-24-s0004.xlsx Primers.

ASM does not own the copyrights to Supplemental Material that may be linked to, or accessed through, an article. The authors have granted ASM a non-exclusive, world-wide license to publish the Supplemental Material files. Please contact the corresponding author directly for reuse.
