# Supplementary material for: Distinct mutations emerge in the genome of serotype O foot-and-mouth disease virus during persistence in cattle
Source: J Virol. 2025 Feb 7;99(3):e01422-24. doi: 10.1128/jvi.01422-24 (PMC11915810; doi:10.1128/jvi.01422-24)
Supplement: Figure S1 — Body temperature and vesicular lesions of the animals in the vaccination study. [file jvi.01422-24-s0001.docx]

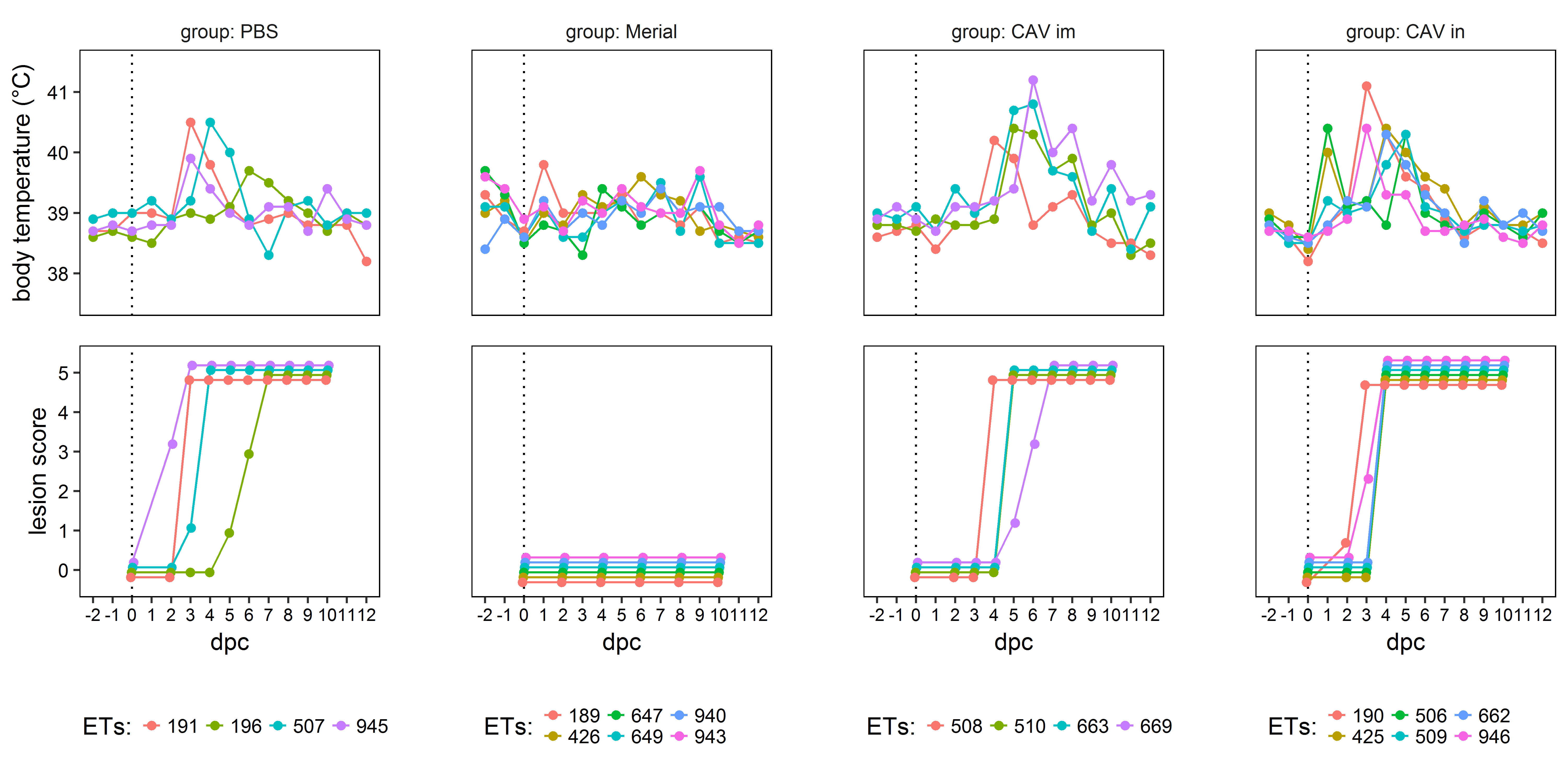


**Supplemental Figure S1**: Body temperature and vesicular lesions of the animals in the vaccination study. One point was awarded for vesicles anywhere on the head and on each foot, for a maximum score of 5.
